# Natural Tooth Pontic: An Instant Esthetic Option for Periodontally Compromised Teeth—A Case Series

**DOI:** 10.1155/2016/8502927

**Published:** 2016-11-22

**Authors:** Rishi Raj, Kriti Mehrotra, Ipshita Narayan, Triveni Mavinakote Gowda, D. S. Mehta

**Affiliations:** Department of Periodontics, Bapuji Dental College & Hospital, Davangere, Karnataka 577004, India

## Abstract

Sudden tooth loss in the esthetic zone of the maxillary or mandibular anterior region can be due to trauma, periodontal disease, or endodontic failure. The treatment options for replacing the missing tooth can vary between removable prosthesis, tooth-supported prosthesis, and implant-supported prosthesis. Irrespective of the final treatment, the first line of management would be to provisionally restore the patient's esthetic appearance at the earliest, while functionally stabilizing the compromised arch. Using the patient's own natural tooth as a pontic offers the benefits of being the right size, shape, and color and provides exact repositioning in its original intraoral three-dimensional position. Additionally, using the patient's platelet concentrate (platelet rich fibrin) facilitates early wound healing and preservation of alveolar ridge shape following tooth extraction. The abutment teeth can also be preserved with minimal or no preparation, thus keeping the technique reversible, and can be completed at the chair side thereby avoiding laboratory costs. This helps the patient better tolerate the effect of tooth loss psychologically. The article describes a successful, immediate, and viable technique for rehabilitation of three different patients requiring replacement of a single periodontally compromised tooth in an esthetic region.

## 1. Introduction

Esthetics and function of the orofacial region are very important aspects of human life, which are affected by anterior tooth loss regardless of personal factors such as age, gender, and level of education, eventually impacting the quality of life [1]. As dentists, we occasionally face daunting conditions that warrant removal of teeth from a high esthetic zone due to trauma, periodontal disease, root resorption, or failed endodontic treatment [2]. Extraction of these teeth mainly leads to esthetic and phonetic difficulties and a functional disability to some extent with pathologic migration. Mostly, such patients either strongly desire to postpone the extraction of their natural teeth or demand immediate management of the esthetic crisis which could adversely affect their social life.

Conventional treatment options available include the removable temporary acrylic prosthesis, resin bonded bridges, and traditional metal and ceramic fixed partial denture (FPD) and amongst the relatively newer options is osseointegrated implant-supported prosthesis [3]. Understanding the patients' cosmetic demands, functional needs, and affordability becomes imperative in delivering the best possible dental service.

In certain clinical scenarios, using an intact natural tooth which is in good clinical condition as pontic for interim duration could offer a plethora of benefits like excellent color, shape, and size match, positive psychological value, minimal cost, and minimum chairside time with no laboratory procedure involved [4]. With recent advancements in adhesive technology and the advent of newer and stronger composite resin materials, it is possible to create a conservative, highly esthetic prosthesis that is bonded directly to teeth adjacent to the missing tooth.

Socket preservation, as a tool for optimizing the preservation of the hard and soft tissue components of the alveolar ridge immediately following tooth extraction, has been accepted as a clinical protocol for more than a decade now [5]. Autologous platelet concentrates are claimed to enhance hard and soft tissue healing due to the considerable amount of growth factors that are released after application in the surgical site [6]. This article describes the clinically replicable technique of socket preservation using platelet rich fibrin (PRF) followed by immediate tooth replacement utilizing the extracted natural tooth as pontic (NTP) to assist the clinicians in providing an esthetically acceptable treatment option.

## 2. Case Series

The case selection criteria for NTP include patient desiring an immediate replacement, patient's unwillingness for any kind of invasive procedure, for example, implant-supported prosthesis, areas with high esthetic demand, and need for a cost-effective treatment protocol.

### 2.1. Case  1

**Figure 1:**
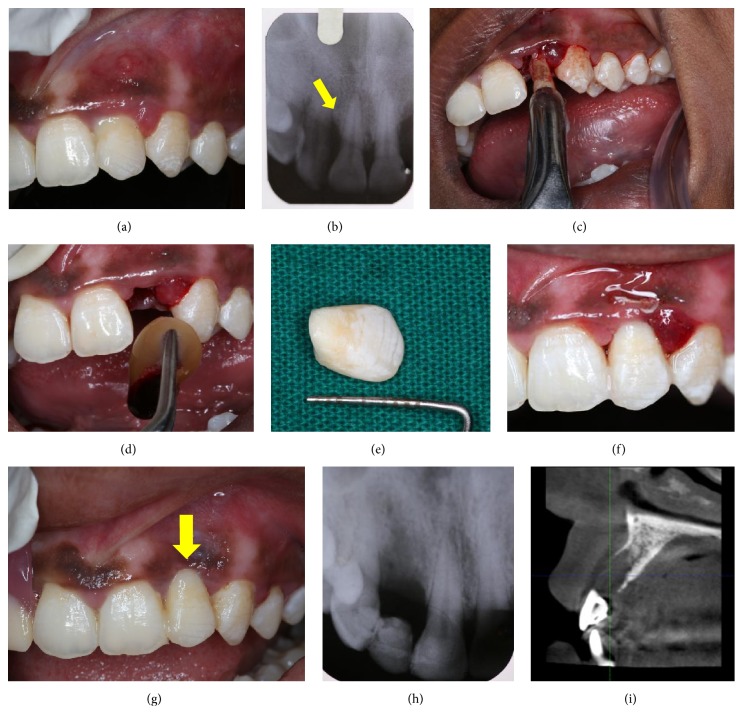
(a) Preoperative. (b) Preoperative radiograph. (c) Extraction of 22. (d) Insertion of PRF. (e) Customized NTP. (f) Immediate postoperative. (g) 1 week postoperatively. (h) 1 week postoperatively. (i) CBCT NTP with respect to 22.

A 22-year-old healthy female patient reported to our department with the chief complaint of mobility in the upper front tooth region along with pus discharge and pain and requested that we provide her with the best treatment possible. Oral and radiographic examination revealed moderate generalized bone loss except for #22 which showed grade III mobility (Figures 1(a) and 1(b)). As the prognosis of #22 was hopeless, different treatment options available to the patient were explained and she chose to use the clinical crown as natural pontic. Extraction of #22 was performed atraumatically under local anaesthesia (Figure 1(c)) and the socket was curetted thoroughly. Then, 5 mL of venous blood was collected from the antecubital fossa of the patient and was immediately transferred to a sterile* (Choukroun's A-PRF)* test tube. The blood was centrifuged at 2700 rpm for 12 minutes, following which PRF obtained was placed in the extraction socket (Figure 1(d)) and stabilized using figure-of-eight suture using 5–0 polyamide* (Ethicon)*.

Thereafter, the length of the natural tooth pontic was determined using periodontal probe and an additional 2 mm was added to compensate for the gingival shrinkage during the healing phase of the extraction site. The natural crown was sectioned from the root using diamond disc to achieve modified ridge-lap shape (Figure 1(e)). Its position was ascertained before bonding, to exclude any occlusal interferences. The pulp chamber was then cleaned, sealed with composite resin* (3M ESPE, Filtek™ Z350)*, and stored in normal saline till replacement. By using floss as template, adequate length of bondable reinforcement ribbon* (Ribbond)* was determined to be inclusive of adjacent teeth. The abutment teeth and the pontic were then etched with 35% phosphoric acid* (3M ESPE)* for 30 seconds, washed, and dried. Thereafter, bonding agent* (Dentsply)* was applied to the etched enamel and cured. A thin layer of composite resin (flowable composite 3M ESPE, Filtek) was placed across the abutment teeth and the pontic. The precut Ribbond fiber was thoroughly wetted by using the bonding agent and placed over the composite and cured. A further layer of composite was placed over the fiber, ensuring that the whole tape was covered by the composite. The excess composite resin was removed and the occlusal interferences were rechecked in protrusion and lateral excursions. Finishing and polishing procedures were carried out by using composite finishing discs and stones. The treatment outcome has been monitored over the last six months and there has been no evidence of any esthetic or functional problems. As the patient desired to undergo implant therapy, CBCT was advised (Figure 1(i)).

### 2.2. Case  2 

**Figure 2:**
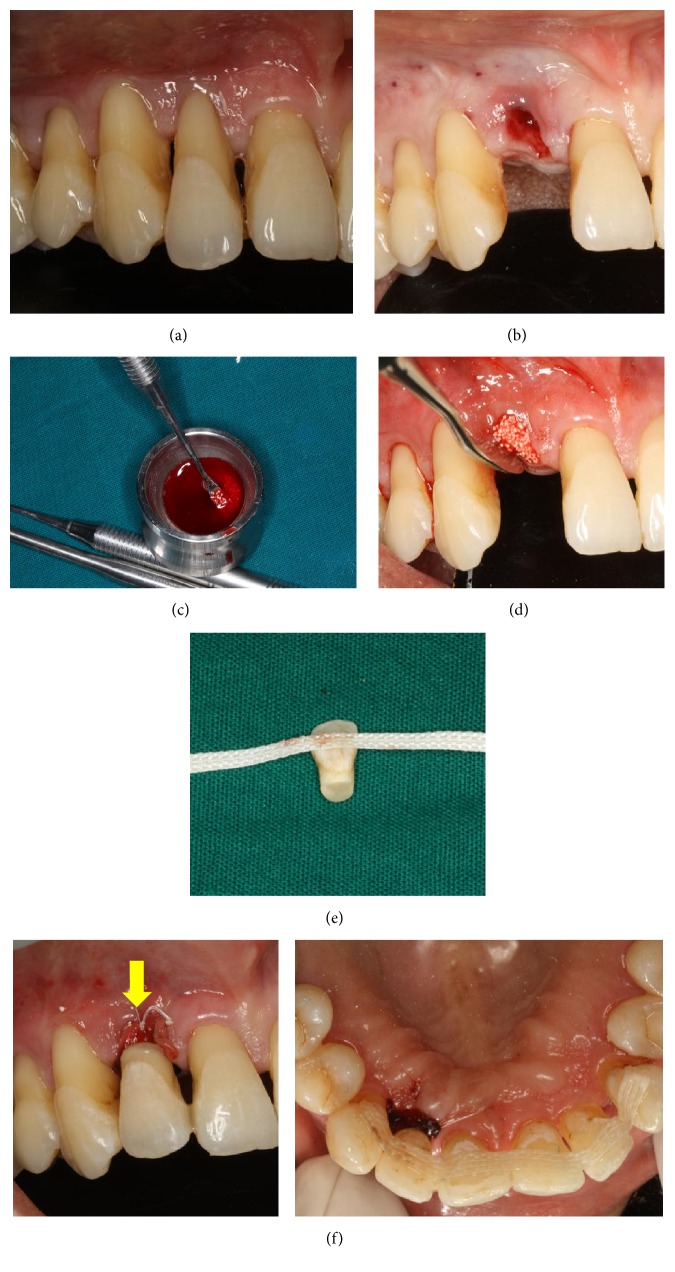
(a) Preoperative. (b) Atraumatic extraction. (c) Preparation of sticky bone. (d) Socket preservation. (e) Ribbond attached to natural crown. (f) Immediate postoperative NTP with respect to 12.

A 35-year-old healthy female patient reported to the department with the chief complaint of mobility of teeth. She was diagnosed with generalized chronic periodontitis with grade III mobility of #12 (Figure 2(a)) which was unsalvageable. Extraction of #12 was performed atraumatically (Figure 2(b)). On inspection, the extraction socket revealed presence of intact bony plate. Hence, we decided to proceed with graft placement. PRF was prepared as described above and mixed with *β*-TCP* (Virchow Co.)* graft material to form a sticky mixture (Figure 2(c)) which was thereafter placed in the extraction site to facilitate socket preservation (Figure 2(d)). PRF plug was used to seal the site and the wound was closed using figure-of-eight (“8”) suture with 5–0 polyamide* (Ethicon)*. The natural crown was used as a pontic in a similar fashion, as has been discussed above (Figures 2(e)-2(f)).

Thereafter, the patient was treated for full mouth periodontal therapy. Posttreatment follow-up was done over the last 9 months and the patient was found to be greatly enthused by the final esthetics and function.

### 2.3. Case  3

**Figure 3:**
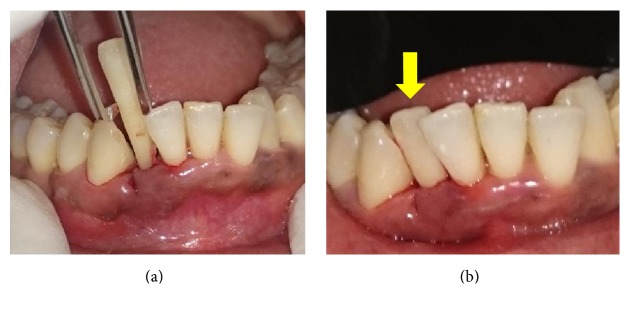
(a) Extraction of 42. (b) NTP with respect to 42.

A 50-year-old healthy female reported to our department with the chief complaint of mobility in the lower front tooth region. Clinical examination revealed grade III mobility in #42. After extraction of #42 (Figure 3(a)), the extraction socket was preserved using PRF and the natural crown was used as pontic (Figure 3(b)).

General oral hygiene instructions were given to all three patients.

## 3. Discussion

The restoration of a smile is one of the most appreciative and gratifying services that a dentist can render. Patients with lost anterior teeth require immediate attention for the restoration of esthetics and function and also the prevention of social trauma. Each of the treatment modalities available has its own benefits and detriments.

Removable temporary partial dentures placed in the immediate postextraction phase are unesthetic due to the presence of clasps that inadequately preserve extraction socket while impeding the healing process and are bulky, hence causing discomfort to the patient and jeopardizing oral hygiene maintenance. For many years, metal-ceramic fixed partial dentures (FPDs) have been the treatment of choice. However, the display of metallic framework is less than esthetically pleasing and also entails aggressive tooth of abutment teeth which increases risk of pulp exposure [7]. Resin retained bridges could provide an alternative owing to limited tooth preparation of the adjacent teeth. However, high frequency of debonding and substantial modification to achieve an acceptable color, size, and shape of the prefabricated acrylic poses a challenge [8].

Postextraction healing and maturation of the bone occur with three-dimensional remodeling even after three months of healing [9]. The clinically growing demand for adequate alveolar housing for implant placement necessitates performing guided bone regeneration (GBR) procedures which would prolong the treatment duration. Immediate implant placement on the other hand is a very case specific protocol. However, some patients reject this therapeutic option, because of either the higher cost or the fear of surgery. Systemic problems may also contraindicate the surgery.

The natural tooth pontic (NTP) technique could be a suitable alternative in such clinical scenario because it is commonly opted for and highly appreciated by the patients for being a single visit technique, not involving any waiting period and temporization. Moreover, cutting of the neighboring teeth can be avoided and is highly cost-effective. Another major advantage of retaining the patient's natural crown is that the patient can better tolerate the effect of tooth loss psychologically [10].

There have been a number of different techniques described in the literature related to restorative dentistry, for splinting teeth using adhesive composite resins, wire, metal mesh, nylon, and so forth bonded to adjacent teeth and adding a natural tooth pontic, denture tooth, or composite resin tooth pontic [7]. The inherent problem with these materials was their inability to be chemically incorporated in composite resin and thus clinical failures were more prevalent due to repeated loading stresses placed on the bridge during normal and paranormal functions. Also, owing to the low fracture strength of the bonded composite resin, pontic might debond unexpectedly and results in an unpleasant social situation. Hence, the challenge was to place a thin but strong bonded composite resin-based single visit bridge using natural tooth as pontic. This was achieved using high-strength polyethylene, bondable, biocompatible, esthetic, easily manipulatable fiber ribbons (Ribbond) that could be embedded into a resin structure [11]. Although reinforced composite materials seem to provide excellent esthetics, some authors do not recommend its use for permanent restoration because of unstable esthetics, increased wear, and liability to plaque accumulation [12]. Clinical studies have shown substantial clinical performance of the fiber reinforced composite (FRC) prosthesis with an overall survival rate of 75% after about 5 years, which is higher than that of the FPDs with metal frameworks [13].

In this case, shape of natural tooth pontic was given as modified ridge-lap pontic with a well-polished and smooth, convex surface that results in pressure-free or mild contact with the alveolar ridge over a very small area for better preservation of the soft tissue health. This particular shape of pontic also helps to give the illusion of the replaced tooth emerging from the gingiva like a natural tooth [4]. Also, the ease of usage and almost no adaptability period as it is with the removable partial denture make it a patient-friendly modality. As with any other treatment modality, this procedure is also associated with a number of limitations like relying on patient's motivation and manual dexterity to maintain oral hygiene around the pontic, limited functional efficiency, irritation to the tongue, and chances of splint breakage. Despite these, studies have shown successful long-term follow-ups of such natural tooth pontics [14, 15].

The use of PRF in the oral cavity has been implicated in different procedures such as extraction socket preservation, intrabony defects, sinus augmentation, and sinus lift procedures for implant placement, bone augmentation, root coverage procedures, and healing in donor site with successful results [16]. The biomaterial acts by releasing high-concentration growth factors to the wound site, thereby stimulating healing and new bone formation [17]. Unlike other socket preservation procedures, the use of PRF is a simple method that requires minimal cost and reduces the need for grafting material. Because it is a completely autologous product, there is absolutely no risk of disease transmission and graft rejection. In the second case, containment of extraction socket allowed socket preservation using PRF and bone graft material.

## 4. Conclusion

All the three patients were satisfied with the esthetic outcome and functioning of this treatment modality, reinforcing its utility as a routine viable option for cases, indicated for extraction of anterior tooth. Natural tooth pontic (NTP) can be placed as interim restoration until an extraction site heals which later if the patient so desires can be replaced by a conventional bridge or an implant. However, appropriate patient selection, their motivation levels, plaque control, and precision during placement of NTP are imperative for its success.
